# How valid is the 2- to 10-day incubation period for cases of Legionnaires’ disease?: A reappraisal in the context of the German LeTriWa study; Berlin, 2016–2020

**DOI:** 10.1017/S0950268823000833

**Published:** 2023-05-29

**Authors:** Ann-Sophie Lehfeld, Markus Petzold, Bonita Brodhun, Walter Haas, Udo Buchholz

**Affiliations:** 1 Department of Infectious Disease Epidemiology, Robert Koch Institute, Berlin, Germany; 2 National Reference Laboratory for Legionella, Institute of Medical Microbiology and Hygiene, University of Technology Dresden, Dresden, Germany

**Keywords:** Incubation period, Legionella, Legionnaires’ disease, LeTriWa study, surveillance

## Abstract

The evidence for the incubation period of Legionnaires’ disease is based on data from a small number of outbreaks. An incubation period of 2–10 days is commonly used for the definition and investigation of cases. In the German LeTriWa study, we collaborated with public health departments to identify evidence-based sources of exposure among cases of Legionnaires’ disease within 1–14 days before symptom onset. For each individual, we assigned weights to the numbered days of exposure before symptom onset, giving the highest weight to exposure days of cases with only one possible day of exposure. We then calculated an incubation period distribution where the median was 5 days and the mode was 6 days. The cumulative distribution reached 89% by the 10th day before symptom onset. One case-patient with immunosuppression had a single day of exposure to the likely infection source only 1 day before symptom onset. Overall, our results support the 2- to 10-day incubation period used in case definition, investigation, and surveillance of cases with Legionnaires’ disease.

## Introduction

The majority of cases of Legionnaires’ disease is sporadic, that is, non-outbreak associated. On epidemiological grounds, three exposure categories are distinguished: travel-associated cases of Legionnaires’ disease (TALD), hospital-associated cases (HALD), and community-acquired cases (CALD). Residential drinking water as well as external, non-residential water sources, such as swimming pools, or workplace exposures may be relevant for CALD [1]. Commonly, when a case of Legionnaires’ disease is reported to the local health department of a European country, an incubation period of 2–10 days is used to identify possible sources of infection [2, 3]. To the best of our knowledge, the duration of the incubation period has only been discussed in three publications [4–6]. In two, the median was pinpointed as 6 days [4, 5], and in one as 7 days [6], while the proportion of cases with an incubation period of more than 10 days was 16% [6], 7% [5], and 5% [4]. Calculating the incubation period is only possible in outbreak investigations with defined short-time exposure [4–6] or alternatively in individual cases when exposure is limited to a single or few days. As part of the Berlin LeTriWa study [1], we identified cases with these characteristics and evaluated the data with regard to the likely incubation period.

## Methods

The Berlin LeTriWa study was a joint project of the Robert Koch Institute, the German Environment Agency, and the Legionella Reference Laboratory in close cooperation with the 12 Berlin district health departments and 15 Berlin hospitals. We collected data from December 2016 to August 2020. The description of the methodology has already been published [1], and only the essential elements are briefly described here.

In the LeTriWa study, we assisted the public health departments in the investigation and surveillance of cases with Legionnaires’ disease (Legionella infection with pneumonia). The aim was to identify evidence-based sources of infection for each case. We conducted a questionnaire inquiring about exposures in the 14 days prior to the day of symptom onset and recorded the exact days when each exposure took place. For all cases of Legionnaires’ disease reported, an additional urine and – if possible – deep respiratory sample of patients were taken and sent to the Legionella Reference Laboratory for typing. In the household of cases we took five standardised water and biofilm samples from the faucet and shower in the bathroom as well as samples from other residential drinking water and non-drinking water sources, if applicable. In addition, we sampled water (and biofilm, if applicable) from other relevant sources, in particular external, non-residential water sources, if possible. Samples were analysed by the laboratory of the German Environment Agency for the presence (and concentration) of Legionella, and isolates were sent to the Legionella Reference Laboratory for typing. We divided cases into two groups: *group 1* were community-acquired cases, eligible to participate in a case–control study and consented to participate. We conducted a case–control study to determine risk factors for cases of Legionnaires’ disease and recruited hospital and age-group matched controls for each case. We asked the controls with the same questionnaire and took the same five standard household samples. *Group 2* cases were either CALD, but did not consent to take part in the case–control study, or were HALD or TALD. These cases were interviewed by the public health department.

We used these pieces of information to identify the likely source of infection for cases. In about 20% of the cases, the patients’ and environmental strains matched with respect to monoclonal antibody (MAb) type, MAb subtype, or sequence type. Because statistical analysis comparing results of case-patients’ standard household water samples with that of simultaneously recruited control persons yielded a strong association with the occurrence of Legionella strains reacting with MAb type 3/1 (Dresden panel) [7], we assumed *microbiological evidence* for a given source of infection as causative when:

1. no information was available on the patient’s MAb type and the potential infection source contained a MAb 3/1-positive strain OR
2. the patient’s strain and that of the potential source did not contradict each other regarding MAb type, MAb subtype, or sequence type.

In addition, and in keeping with den Boer [8], we applied another type of evidence (*cluster evidence*) when a certain exposure was mentioned by at least 2 cases, that is, if – within a two-year period – at least 2 cases were exposed to the same source, this source was attributed to the respective cases.

If, at the same time, several sources had the same type of evidence, we attributed the probable infectious water source on an individual basis.

For the analysis presented here, we included CALD, TALD, and HALD who were probably infected via an external, non-residential water source based on microbiological evidence or cluster evidence, or who were associated with a travel accommodation or hospital stay based on microbiological evidence or cluster evidence. Cases were excluded if the exact days of exposure could not be remembered or if they were exposed to the source for more than 6 days. We set the cut-off at 6 days to strike a balance between the cases with a lesser number of days of exposure and the cases with a higher number of days of exposure to the likely source of infection. To compare the cases who had only 1 day of exposure with the cases who had multiple days of exposure, the individual exposure days of each case were weighted. The total weight of all exposure days combined for each case was always 6. Table 1 shows schematically, using a fictitious example, how the exposure days were weighted. In this fictitious example, case 1 was exposed to the probable source of infection only on day 5 before symptom onset; that day received a weight of 6. Case 2 had 3 days of exposure, on days 4, 8, and 12. Those 3 days each received a weight of 2, resulting in a total weight of all exposure days of 6. We summed the weights of all cases over all exposure days from day 1 to day 14 before symptom onset, respectively, and presented them as a weighted frequency distribution, which could also be displayed as a cumulative probability function (using relative proportions). Information on predisposing risk factors was known only for some case-patients.

**Table 1.** Scheme for weighting days of exposure for cases with different numbers of exposure days to the probable source of infection. A fictitious example is shown

	Day before symptom onset														Number of exposure days	Total weight of all exposure days
Case	1	2	3	4	5	6	7	8	9	10	11	12	13	14		
Case 1					6										1	6
Case 2				2				2				2			3	6

## Results

**Figure 1.**
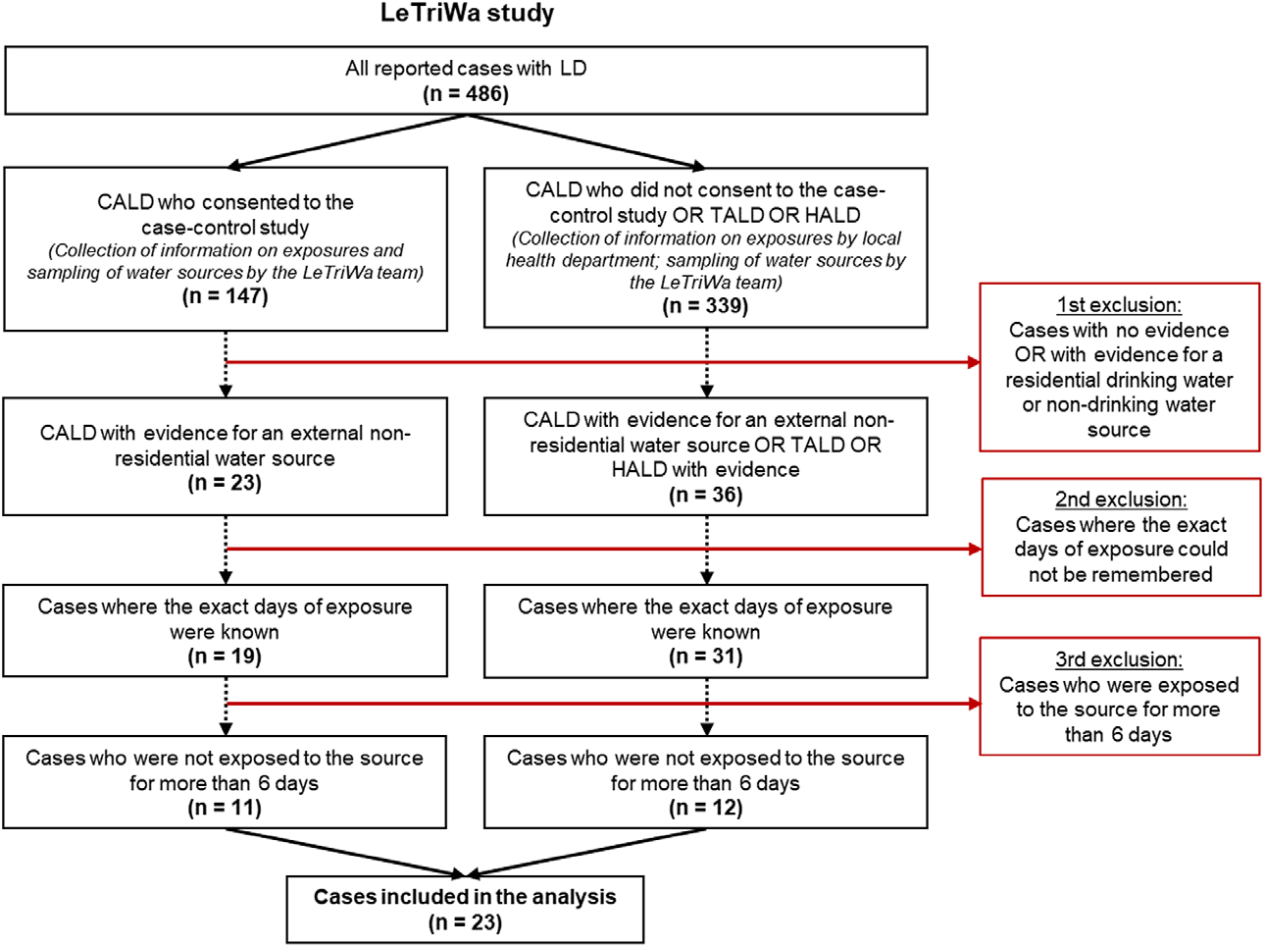
Breakdown of cases included in the analysis. Left side: Only CALD who consented to the LeTriWa case–control study (case group 1). Right side: TALD and HALD as well as CALD who did not consent to the case–control study (case group 2). *Note:* *N* = Number of cases remaining after exclusion (red boxes). LeTriWa study 2016−2020; Berlin, Germany. CALD = community-acquired cases of LD; HALD = hospital-associated cases of LD; LD = Legionnaires’ disease; TALD = travel-associated cases of LD.

A preliminary analysis of a subset of these data was published in German [9]. Between December 2016 and August 2020, a total of 486 cases of Legionnaires’ disease were reported to the Robert Koch Institute. We were able to assign a total of 59 cases (31 CALD, 16 HALD, and 12 TALD) to a probable source of infection based on microbiological evidence and/or cluster evidence for an external, non-residential source, a travel accommodation, or a hospital stay (Figure 1; total number of cases after the first exclusion). Of these, we excluded 9 cases because the exact days of exposure could not be remembered and another 27 cases because they were exposed for more than 6 days to the probable source of infection. This left 23 cases (14 CALD, 5 TALD, and 4 HALD) for analysis who were exposed to a specific water source on at least 1 day and a maximum of 6 days (Table 2). Restricting the cases to those with a maximum of 4 days of exposure showed similar results.

**Table 2.** Distribution and number of exposure days per case of cases with evidence of a probable source of infection within the queried 14-day exposure period before symptom onset. For each case, the weighted exposure days are provided, for example, case no. 1 had only 1 exposure day on day 1, so that day received a weight of 6, whereas case no. 7 had 2 exposure days on day 6 and 13, so each of both days received a weight of 3. For each case, the sum of the weighted exposure days gives a total weight of 6. In addition, the 2- to 10-day incubation period is framed, which is usually queried by the public health departments. The three columns on the right list the patient strain, environmental strain, and the type of evidence for the probable source of infection. LeTriWa study 2016−2020; Berlin, Germany

Case no.	Case group	Exposure categories	Exposure period queried in LeTriWa (1–14 days before symptom onset)														No. of exposure days		Patient strain	Environmental strain of the probable source of infection	Type of evidence
			1	2	3	4	5	6	7	8	9	10	11	12	13	14	2–10 days before symptom onset	1–14 days before symptom onset			
1	2	CALD	6														0	1	Lp1	Lp1, MAb 3/1+, Knoxville, ST182	mb
2	2	CALD				6											1	1	Lp1, MAb 3/1+	Lp1, MAb 3/1+, Knoxville, ST182	mb and cluster
3	2	CALD					6										1	1	Lp1	Legionella negative	cluster
4	1	CALD						6									1	1	Lp1, MAb 3/1+	Lp1, MAb 3/1+, Knoxville, ST182	mb
5	1	CALD						6									1	1	Lp1, MAb 3/1+, Knoxville, STnon182^a^	Lp1, MAb 3/1+, Knoxville, ST182	cluster
6	1	CALD									6						1	1	Lp1, MAb 3/1+, Knoxville	Lp1, MAb 3/1+, Knoxville, ST182	mb
7	1	CALD						3							3		1	2	Lp1, MAb 3/1+, Knoxville	Lp1, MAb 3/1+, Knoxville, ST182	mb
8	1	CALD	3							3							1	2	Lp1, MAb 3/1+	Lp1, MAb 3/1+, Knoxville, ST182	mb and cluster
9	1	CALD							3							3	1	2	Lp1	Lp1, MAb 3/1+, Knoxville, ST182	mb and cluster
10	2	TALD					3	3									2	2	Lp1	Legionella negative	cluster
11	2	HALD									3	3					2	2	Lp1, MAb 3/1+, Knoxville, ST182	Lp1, MAb 3/1+, Knoxville	mb and cluster
12	2	HALD		3	3												2	2	Lp1	Not sampled or unknown	cluster
13	1	CALD				2	2	2									3	3	Lp1, MAb 3/1+, Knoxville, ST182	Lp1, MAb 3/1+, Knoxville, ST182	mb
14	1	CALD				1.5		1.5					1.5		1.5		2	4	Lp1, MAb 3/1+, Knoxville	Lp1, MAb 3/1+, Knoxville, ST182	mb
15	1	CALD		1.5			1.5				1.5			1.5			3	4	Lp1	Lp1, MAb 3/1+, Knoxville, ST510	mb and cluster
16	2	HALD						1.5	1.5	1.5	1.5						4	4	Lp1, MAb 3/1+	Legionella positive, species unknown	cluster
17	1	CALD	1.2	1.2			1.2	1.2	1.2								4	5	Lp1	Lp1, MAb 3/1+, Knoxville, ST182	mb and cluster
18	2	TALD							1.2	1.2	1.2	1.2	1.2				4	5	Lp1	Lp1, MAb 3/1+, Knoxville	mb
19	2	TALD	1.2	1.2	1.2	1.2	1.2										4	5	Lp1, MAb 3/1+	Not sampled or unknown	cluster
20	2	HALD	1.2	1.2	1.2	1.2	1.2										4	5	Lp1, MAb 3/1+, Knoxville	Not sampled or unknown	cluster
21	2	TALD			1.2	1.2	1.2	1.2	1.2								5	5	Lp1	Lp1, MAb 3/1+, Benidorm	mb
22	2	TALD									1	1	1	1	1	1	2	6	Lp1	Not sampled or unknown	cluster
23	1	CALD	1	1	1	1	1	1									5	6	Lp1, MAb 3/1+, Knoxville	Lp1, MAb 3/1+, Knoxville, ST9	mb
				usual incubation period (2–10 days before symptom onset)																	

Abbreviations: CALD, community-acquired cases of Legionnaires’ disease; HALD, hospital-associated cases of Legionnaires’ disease; Lp1, *Legionella pneumophila* serogroup 1; MAb, monoclonal antibody; ST, sequence type; mb, microbiological; TALD, travel-associated cases of Legionnaires’ disease.

a In case 5, the ST was unknown, but ST182 was excluded based on the (partially) determined allele sequence. The water source was nevertheless attributed to the case because of cluster evidence.

Of the 23 cases, 9 cases were attributed to a probable source of infection based on microbiological evidence, 8 cases were attributed based on cluster evidence, and 6 cases had both microbiological and cluster evidence (right column in Table 2). The number of exposure days to the probable source of infection within the queried 14-day exposure period varied between 1 and 6 days for the 23 cases. Table 2 shows the distribution of exposure days before symptom onset (and their respective weight) for each case for the queried 14-day exposure period as well as for the usually used incubation period of 2–10 days (sorted by number of exposure days (fourth column from right)). There were 6 cases exposed to the probable source of infection on only 1 day in the 14 days before symptom onset (cases 1–6). Considering only the period of 2–10 days before symptom onset, 35% of the cases (8 of 23) had a single day of exposure (fifth column from right; cases 2–9). For these 8 cases, this was 3 times on day 6 and once each on days 4, 5, and 7–9.

**Figure 2.**
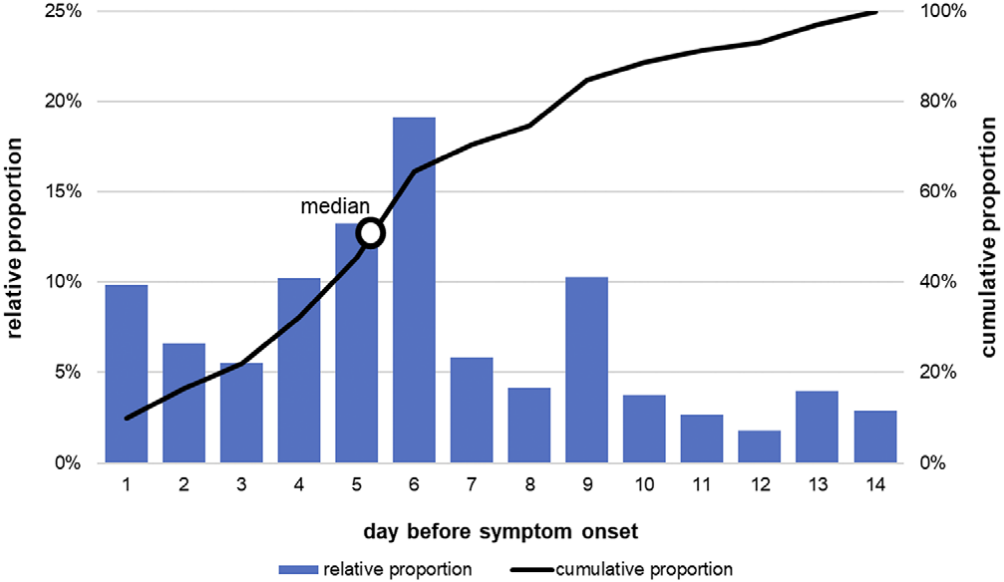
Weighted distribution of exposure days (incubation period distribution) within the queried 14-day exposure period before symptom onset expressed as relative proportions (bars) and cumulative proportions (line) (number of cases = 23; multiple exposure days possible). LeTriWa study 2016−2020; Berlin, Germany.

Figure 2 shows the distribution of the weighted exposure days before symptom onset from Table 2 (incubation period distribution; multiple exposure days possible) as well as the cumulative distribution. The mode – that is, the most frequently mentioned day – is 6 days and the median is 5 days (using only cases who had microbiological AND cluster evidence for the probable source of infection (n = 6), the median was 6 days). Approximately two-thirds (61%; 14 of 23) of the cases were exposed to the probable source of infection on day 5 or 6 before symptom onset (Table 2). The cumulative distribution reached 89% by day 10 before symptom onset, and 11% of the weighted incubation period distribution lies between days 11 and 14. Overall, there were 11 cases with exposure to the probable source of infection outside of the 2–10 days (i.e., on day 1 or days 11–14), and of these, 6 cases were exposed after the 10-day period, but 10 of the 11 cases were also exposed to the probable source of infection within the usual incubation period of 2–10 days (Table 2). In only 1 case did we detect exposure to the probable source of infection exclusively outside the usually queried 2- to 10-day incubation period, namely on day 1 before symptom onset (case 1 in Table 2). This case had an immunosuppression as underlying disease. Cases 4, 10, and 14 also were immunosuppressed and had possible incubation periods of 6 days, 5 or 6 days, and 4, 6, 11, or 13 days, respectively (Table 2).

## Discussion

The results of the LeTriWa study support the assumption of the incubation period for cases of Legionnaires’ disease used in surveillance and the current approach of identifying possible sources of infection within the 2–10 days before symptom onset. The result of the median incubation period of 5 days differs only by 1 [4, 5] or 2 [6] days from other international publications based on outbreak investigations.

We were also able to identify exposures outside the usually used incubation period. About 11% of the weighted distribution of incubation period lay after day 10 (i.e., between days 11 and 14), which is also in good agreement with the international publications mentioned above, where 5−16% of cases had an incubation period of more than 10 days [4–6]. In relation to the cases, about a quarter of cases (26%; 6 of 23) were also exposed after day 10 before symptom onset. On the one hand, these were cases whose exposures were due to a regular activity, for example, cases who were exposed once a week to the probable source of infection such as a swimming pool. On the other hand, these were travel-associated case-patients who had exposure to the travel accommodation more than 10 days before symptom onset. However, all of these 6 cases with a possible incubation period longer than 10 days were also exposed during the usually used incubation period. We identified only 1 case who was exposed to the probable source of infection exclusively on day 1 before symptom onset, that is, outside the 2- to 10-day incubation period (case 1 in Table 2). In this case, MAb 3/1-positive strains were identified in the probable external, non-residential source of infection (microbiological evidence), whereas MAb 3/1-negative strains were identified in the residential drinking water. This case had a strongly predisposing underlying disease, which possibly led to a shortening of the incubation period. The other three cases with immunosuppression had an incubation period of 6 days, 5 or 6 days, and 4, 6, 11, or 13 days, respectively (cases 4, 10, and 14 in Table 2). In the literature, few case studies with immunosuppression can be found; an Italian case study reported an incubation period of at least 14 days in a patient with a kidney transplant [10].

Another finding is that about one-third (35%) of all cases and more than half of CALD (58%) had only 1 day of exposure within the 2- to 10-day incubation period. For practical purposes, this means that cases of Legionnaires’ disease should be queried as completely as possible, even if the exposure was apparently transient or short term. For external, non-residential water sources, we identified mainly workplace exposures (including exposures only to faucets of hand-washing sinks) or exposures to showers in swimming pools as a probable source of infection [1].

A limitation of the present analysis is that although we have already queried a longer time period of 14 days before symptom onset, an incubation period of up to 19 days was found in a large outbreak in the context of a flower show in the Netherlands [6]. Incubation periods of up to 26 days have also been reported for individual cases in outbreaks [11, 12]. Therefore, it is theoretically possible that we missed relevant exposures longer than 14 days before symptom onset. Another limitation is that infectious sources have been tested in various intervals after the day of infection, and thus, if exposure was transient or Legionella concentration was low, it might have been missed. In addition, not all possible infectious sources were or can possibly be tested. This applies, for example, for the hand-wash sinks used.

In summary, our results support the 2- to 10-day incubation period used in the investigation and surveillance of cases of Legionnaires’ disease. In immune-compromised cases, it should also be considered to query exposures a day before symptom onset.

## Data Availability

The authors declare that the data supporting the findings of this study are available within the article.
